# The Impact of Clinical, Biochemical, and Echocardiographic Parameters on the Quality of Life in Patients with Heart Failure with Reduced Ejection Fraction

**DOI:** 10.3390/ijerph182312448

**Published:** 2021-11-26

**Authors:** Marta Kałużna-Oleksy, Filip Sawczak, Agata Kukfisz, Helena Krysztofiak, Magdalena Szczechla, Marta Wleklik, Katarzyna Przytarska, Magdalena Dudek, Alicja Nowak, Ewa Straburzyńska-Migaj, Bartosz Uchmanowicz

**Affiliations:** 1 1st Department of Cardiology, University of Medical Sciences in Poznan, 61-848 Poznan, Poland; marta.kaluzna@wp.pl (M.K.-O.); fsawczak@gmail.com (F.S.); agata.kukfisz@gmail.com (A.K.); szczechlamagdalena@gmail.com (M.S.); katarzyna.przytarska@gmail.com (K.P.); magdamroz8@gmail.com (M.D.); alicja.nowak@skpp.edu.pl (A.N.); ewa.straburzynska-migaj@skpp.edu.pl (E.S.-M.); 2 Faculty of Health Sciences, Wroclaw Medical University, 50-367 Wroclaw, Poland; marta.wleklik@umed.wroc.pl (M.W.); bartosz.uchmanowicz@umed.wroc.pl (B.U.)

**Keywords:** quality of life, health-related quality of life, heart failure, heart failure with reduced ejection fraction

## Abstract

Despite significant advances in HF diagnosis and treatment over the recent decades, patients still characterize poor long-term prognosis with many recurrent hospitalizations and reduced health-related quality of life (HRQoL). We aimed to check the potential relationship between clinical, biochemical, or echocardiographic parameters and HRQoL in patients with HF with reduced ejection fraction (HFrEF). We included 152 adult patients hospitalized due to chronic HFrEF. We used the WHOQoL-BREF questionnaire to assess HRQoL and GNRI to evaluate nutritional status. We also analyzed several biochemical parameters and left ventricle ejection fraction. Forty (26.3%) patients were hospitalized due to HF exacerbation and 112 (73.7%) due to planned HF evaluation. The median age was 57 (48–62) years. Patients with low somatic HRQoL score had lower transferrin saturation (23.7 ± 11.1 vs. 29.7 ± 12.5%; *p* = 0.01), LDL (2.40 (1.80–2.92) vs. 2.99 (2.38–3.60) mmol/L; *p* = 0.001), triglycerides (1.18 (0.91–1.57) vs. 1.48 (1.27–2.13) mmol/L; *p* = 0.006) and LVEF (20 (15–25) vs. 25 (20–30)%; *p* = 0.003). TIBC (64.9 (58.5–68.2) vs. 57.7 (52.7–68.6); *p* = 0.02) was significantly higher in this group. We observed no associations between HRQoL and age or gender. The somatic domain of WHOQoL-BREF in patients with HFrEF correlated with the clinical status as well as biochemical and echocardiographic parameters. Assessment of HRQoL in HFrEF seems important in everyday practice and can identify patients requiring a special intervention

## 1. Introduction

The prevalence of heart failure (HF) is still increasing [1] and is estimated to be approximately 1–2% in adults in developed countries. Moreover, in patients above 70 years of age, HF incidence rises to ≥10%, making it a growing healthcare burden [2,3,4,5]. The population of patients with HF is enormous. Over 5 million Americans and 15 million Europeans suffer from HF [6,7,8,9], and in total, an estimated 26 million people suffer from HF worldwide [1]. Despite significant advances in HF diagnosis and treatment over the recent decades, HF patients still characterize poor long-term prognosis [1,10] with high rates of recurrent HF hospitalizations accompanied, however, by slightly lower mortality rates [1]. Although the prevalence of HF with reduced ejection fraction (HFrEF) has decreased in recent years, the mortality rate is still high in this group of HF patients [11]. The number of hospitalizations due to HF is still rising, and it has tripled over the last three decades. HF hospitalizations pose a significant problem since more than one-third of patients are re-hospitalized or die within 90 days after initial discharge [12]. HF is responsible for a large proportion of deaths as well as for diverse morbidities that lead to reduced quality of life (QoL) in this population [9]. HF patients might develop severe disease symptoms and have a fatal prognosis of deterioration of health status and higher risks of recurrent hospitalizations [4] and depression [13,14]. All these factors lead to a well-documented decreased QoL [15]. Furthermore, more patients with HF would rather live better than longer [16].

According to the World Health Organization, health-related quality of life (HRQoL) is “an individual’s perception of their position in life in the context of the culture and value system in which they live, and in relation to their goals, expectations, standards, and concerns” [17]. This definition explains why HRQoL should be assessed using self-reported questionnaires. Chronic diseases, as HF is, decrease HRQoL [15], but there are also other important factors, including social, environmental, and psychological ones. This is why some questionnaires divide HRQoL into domains to understand the influence of a disease better [17]. HRQoL reflects physical, psychological, including emotional and cognitive, and social functioning [18]. HRQoL and its perception in HF and other diseases vary significantly between countries and regions [19,20,21,22,23]. On the other hand, the international study concerning congenital heart disease revealed that HRQoL depended only on patient characteristics, not the cultural milieu [24]. 

The negative impact of HF on HRQoL is well known; therefore, improving HRQoL is one of the main goals of HF management strategies [10]. HRQoL is the therapy target and one of the approved endpoints of clinical trials. Measuring QoL in HF patients is also essential because it can be improved by various interventions such as long-term moderate exercise training [25,26,27], implanting a left ventricular assist device (LVAD) [28,29], or atrial fibrillation ablation [30]. Tackling the issue of self-reported HRQoL is also essential because it could be inappropriate to measure HRQoL without using a proper instrument. Some studies revealed a significant difference between patient’s self-rated HRQoL and the assessment made by the family and healthcare professionals [31]. In patients with advanced HF who qualified for mechanical circulatory support intervention, HRQoL may also influence the treatment decision. Patients with lower HRQoL had more benefits from LVAD implantation compared to optimal medical management. This was contrary to patients with higher HRQoL who showed no benefits from LVAD versus optimal medical management [32].

We aimed to analyze several clinical, biochemical, and echocardiographic parameters in patients with HFrEF. To the best of our knowledge, this is the first study assessing the influence of clinical, biochemical, and echocardiographic parameters on HRQoL in HFrEF patients.

## 2. Materials and Methods

### 2.1. Participants

A total of 152 adult patients hospitalized at the 1st Department of Cardiology, Poznan University of Medical Sciences, between January 2019 and December 2019 due to stable or exacerbated chronic HFrEF were enrolled in this single-center, prospective, observational study. The disease classification was done according to the International Classification of Diseases (ICD–10) for the diagnosis of HF (I50). The inclusion criteria were: (1) admission due to chronic HF (ICD–10 code for main diagnosis I50); (2) age ≥18 years; (3) HF history longer than three months; (4) left ventricular ejection fraction (LVEF) <40%; (5) signing the informed consent form. All patients who met the above inclusion criteria were enrolled in our study. Subjects in a severe general condition and those with severe acute infections or active neoplastic disease as well those with cognitive function impairment not allowing to fill out the questionnaires, were excluded.

The research was conducted in compliance with the Declaration of Helsinki and accepted by the Ethics Committee of Poznan University of Medical Sciences (approval code 477/19).

### 2.2. Clinical Assessment

The medical history with a particular interest in comorbidities, HF etiology, and prescribed medicines was taken on admission. We assessed patients according to the New York Heart Association (NYHA) functional classification as indicated in the European Society of Cardiology (ESC) guidelines [10]. Blood pressure (BP), heart rate (HR), height, and body mass were measured during physical examination. The following formula was used to calculate body mass index (BMI): BMI = weight (kg)/(height (m))^2^ [33]. In fasting blood samples, the following parameters were analyzed: complete blood count, B-type natriuretic peptide (BNP), lipid profile, creatinine, fasting glucose, serum protein and albumin, thyroid hormones, electrolytes (sodium, potassium), and iron metabolism parameters, i.e., iron level, transferrin saturation, total iron-binding capacity (TIBC) and ferritin. LVEF was measured using the Simpson method [34]. The Geriatric Nutritional Risk Index (GNRI) is a tool used to define the level of nutritional risk based on only two variables: serum albumin level and BMI. GNRI for each patient was calculated using the formula (1.489 × serum albumin [g/L]) + (41.7 × body weight/ideal body weight (IBW) [kg]). IBW was calculated as follows: IBW = height^2^ [m] × 22 [35].

### 2.3. Quality of Life Assessment

The use of validated instruments to assess HRQoL remains unclear and limited to clinical trials, with minimal guidance on the practical assessment of HRQoL outside this setting [36]. Therefore, in this study, the authors used the World Health Organization’s Quality of Life Instrument–Short Version (WHOQoL-BREF) to evaluate the HRQoL of patients with HFrEF. It was designed and developed to allow easier and faster assessment of HRQoLmore applicable in common practice than the WHOQoL instrument based on 100 questions divided into six domains and 24 sub-domains [17]. WHOQoL-BREF consists of 26 questions; 24 questions are divided into four domains: physical (somatic), psychological, social, and environmental. There are two additional questions about the self-rated QoL and satisfaction from health status [37]. The score of every domain is transformed into the number ranging between 0 (worst possible QoL) and 100 (best possible QoL) [37]. It was validated and showed acceptable reliability to substitute the original form [38]. The authors used the Polish version of WHOQoL-BREF. The acceptable internal consistency was demonstrated with Cronbach’s alpha coefficients greater than 0.70 for all domains except for the social domain [39].

### 2.4. Statistical Analysis

To assess the relation of the somatic domain score with the parameters studied, patients were divided according to their somatic HRQoL into three subgroups: subjects with the best somatic HRQoL (group 1), with intermediate results (group 2), and with the worst somatic HRQoL (group 3). Then, we compared analyzed parameters between the first and third groups using Student *t*-and U-Mann tests (depending on the presence of normality and variance compliance) for continuous variables and the Pearson Chi2 test for categorical ones. Continuous data values are presented as mean ± standard deviation (SD) or median (interquartile range) according to presence of normal distribution and categorical ones as number (%). *p*-value <0.05 was considered significant. All statistics were performed using Statistica version 13.3 software (StatSoft, now Tibco, Palo Alto, CA, USA).

## 3. Results

The study sample consisted of 152 consecutive patients with HFrEF, 124 (81.6%) of whom were men. 40 (26.3%) patients were hospitalized due to chronic HF (CHF) exacerbation and 112 (73.7%)-due to planned CHF evaluation. The median age was 57 (48–62) years. In the study population, 2% of the patients presented NYHA class I, 40.8% NYHA class II, 46.7% NYHA class III, and 10.5% NYHA class IV. The median LVEF was 22 (20–30)%. At the time of enrollment, 96.7% of patients received beta-blockers, 64.5% *angiotensin-converting enzyme inhibitors* (ACEI) or angiotensin receptor blockers (ARB), 24.3% angiotensin receptor-neprilysin inhibitor (ARNI), and 86.8% mineralocorticoid receptor antagonists (MRA). Median scores of WHOQoL-BREF domains were as follows: somatic 50 (42.9–57.1), psychological 66.7 (58.3–70.8), social 75.0 (66.7–91.7) and environmental 71.9 (62.5–81.2). Total HRQoL calculated as the sum of all four domains was 265.2 (239.7–285.7). The baseline characteristics of the study group are described in Table 1.

According to the results of the somatic domain in WHOQoL-BREF questionnaire patients were divided into three subgroups: 47 patients (30.9%) had >55 points (group 1), 47 (30.9%) had 55–50 points (group 2), and 58(38.2%) had <50 points (group 3).

We compared patients with the highest score in the somatic domain of the WHOQoL-BREF questionnaire with those with the lowest score (group 1 vs. group 3) (Table 2). We observed no significant differences in gender, age, HF etiology (ischemic or non-ischemic) as well in comorbidities’ rates between the groups.

**Figure 1:**
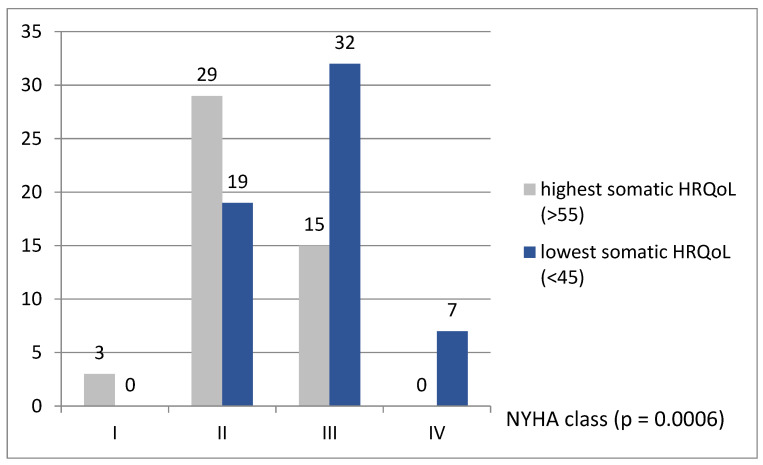
NYHA class in patients with highest and lowest somatic HRQoL score.

Patients with the lowest somatic HRQoL score (group 3) lower BMI (27.1 (23.8–31.7) vs. 28.7 (26.0–32.3) kg/m^2^), but it was not statistically significant (*p* = 0.08). The NYHA class on admission was higher in patients with the lowest HRQoL score than those with the highest HRQoL score (Figure 1). NYHA classes III–IV were more likely observed in group 3 (57.3% vs. 31.9%; *p* = 0.0003). Patients from the group with the highest somatic domain of QoL had also higher scores in all other measured domains of WHOQoL-BREF: psychological (66.7 (62.5–79.2) vs. 62.5 (58.3–70.8); *p* = 0.004), social (83.3 (75.0–100) vs. 66.6 (58.3–83.3); *p* < 0.0001) and environmental (75.0 (65.6–87.5) vs. 68.8 (59.4–75.0); *p* = 0.001). Moreover, total HRQoL (288.2 (267.6–317.3) vs. 244.6 (219.5–263.1); *p* < 0.0001) was better in patients with the highest score in the somatic domain.

We also observed significant differences in biochemical parameters when we compared group 3 (worst quality of life) with group 1: uric acid level was higher (492 (383–559) vs. 424 (318–534) µmol/L; *p* = 0.03), while potassium (4.21 ± 0.43 vs. 4.45 ± 0.39 mmol/L; *p* = 0.004), total cholesterol (4.14 (3.20–4.79) vs. 4.61 (3.82–5.42) mmol/L; *p* = 0.01), triglycerides (TG) (1.18 (0.91–1.57) vs. 1.48 (1.27–2.13) mmol/L; *p* = 0.006) and LDL (2.40 (1.80–2.92) vs. 2.99 (2.38–3.60) mmol/L; *p* = 0.001) levels were lower. There was higher BNP level in the group with lowest somatic HRQoL (578.3 (209.6–1124) vs. 326.2 (162.2–687.9) pg/mL), but this difference was not statistically significant (*p* = 0.06). Moreover, we analyzed the iron deficiency profile. We revealed lower transferrin saturation (23.7 ± 11.1 vs. 29.7 ± 12.5 %; *p* = 0.01) and higher TIBC (64.9 (58.5–68.2) vs. 57.7 (52.7–68.6); *p* = 0.02) in the group with lower somatic HRQoL score. Iron concentration was lower in that group (15.1 ± 6.8 vs. 17.6 ± 7.0 μmol/L), but it was not statistically significant (*p* = 0.08),

LVEF was significantly lower in patients with a low somatic HRQoL score (20 (15–25) vs. 25 (20–30) %; *p* = 0.003).

We also evaluated the nutritional status using the GNRI assessment tool. Patients with low somatic HRQoL also presented lower GNRI (112.3 ± 11.4 vs. 117.8 ± 12.4; *p* = 0.045), although most patients were qualified to the no-risk group with the GNRI higher than 98 (Table 2).

## 4. Discussion

Increasing HF incidence poses a substantial social problem, and patients with HFrEF are a specific group characterized by poor prognosis. HF is a chronic disease; therefore, HRQoL is very important since patients must live with the disease for many years. Some studies showed that for certain patients, HRQoL is more important than the life span [16]. This was proved in a study with HFrEF patients and the mean LVEF of 33 ± 12% [16], i.e., in a group which we also analyzed in our research (median LVEF 22 (20–30)). Several factors impact HRQoL, including both somatic as well as psychological factors [40,41,42]. The primary treatment goal in HFrEF patients is complex and should optimize their health status (i.e., symptoms, functions, and QoL) [9]. The Minnesota Living with Heart Failure Questionnaire (MLHFQ) and the Kansas City Cardiomyopathy Questionnaire (KCCQ), the two most widely used tools to measure HRQoL in HF, were developed in patients with HFrEF [43,44,45]. However, there is no single tool assessing HRQoL in HFrEF patients that would be recommended as a reference tool in this population. In our study, we have chosen the WHOQoL-BREF questionnaire as a simple in use but simultaneously a comprehensive tool.

There are multiple factors in HF which impact HRQoL. Patients with higher NYHA class had lower HRQoL [15,46,47] than patients in NYHA class I or II. We also reported this in our study. In the general population, older women reported worse QoL and a higher level of disability than men [23]. In some studies, women with HFrEF or with HF with preserved ejection fraction (HFpEF) reported worse QoL and a higher level of disability in a self-assessment tool compared to male patients [13,48]. At the same time, in the other, there was no correlation [46] or it was observed only in general HRQoL and not in HF-specific (not related just to HF but also to other comorbidities) [47]. In our study, the lower somatic domain score in the WHOQoL-BREF questionnaire was observed in patients with higher NYHA class, but we observed no difference according to age and gender.

### 4.1. Natriuretic Peptides

High BNP or NT-proBNP are well documented as connected with a worse prognosis in HF. They are predictors of more frequent hospitalizations [49], rehospitalizations [50], deaths [51], and more severe symptoms [52]. Higher BNP or NT-proBNP levels are associated independently with the worse overall and physical domain of QoL [47,53,54]. However, Faxén et al. used other QoL questionnaires, the generic EQ-5D3Land MLHFQ, based on differently determined domains [47]. Moreover, Faxén et al. also enrolled patients with stable HFpEF, and this was contrary to our study that comprised solely of patients with HFrEF. Interestingly, Allen et al. used the KCCQ to assess QoL [53]. Moreover, the group included in that study was similar to our group, i.e., all patients had LVEF ≤ 40% [53]. Furthermore, Hoekstra et al. applied three questionnaires to assess QoL [54]. Authors evaluated QoL in three different ways: global well-being (by using the Cantril’ s Ladder of Life), general QoL (by using the Medical Outcome Study 36-item General Health Survey-RAND-36), and disease-specific QoL (measured by MLHFQ).The detail of the questions in the WHOQoL-BREF questionnaire and three questionnaires used by Hoekstra et al. seems to be similar. However, contrary to our study, Hoekstra et al. enrolled both HFpEF and HFrEF patients.

Decrease in BNP level is associated with improved QoL [55], nonetheless premises from many studies suggest that therapy guided on current BNP concentration is economically unreasonable and does not improve the QoL [50,55,56,57,58].

### 4.2. Left Ventricular Ejection Fraction

The correlation between LVEF and the HRQoL in HF patients is not evident, and there are many contrary results. HFpEF patients are reported to have worse HRQoL than those with HFrEF; borderline LVEF patients generally had intermediate parameters [59]. Also, higher LVEF was associated with worse HRQoL when adjusted by sex, BMI, comorbidities, and non-white race [59]. Nevertheless, these studies were heterogeneous and enrolled both HFrEF and HFpEF patients. Other studies suggest no differences between HFpEF and HFrEF and no correlation between HRQoL and LVEF [46,60]. In another study, patients with lower LVEF showed a higher risk of death or severely decreased QoL after 1 and 24 weeks [53]. By contrast, NYHA III class patients with higher LVEF and less severe diastolic dysfunction were associated with better self-reported QoL. That association remained statistically significant after adjustment for age, gender, hypertension, angina pectoris class, nitrate, ACE inhibitors, and diuretics use [61]. In NYHA I and II, the correlation was not statistically significant [61]. In our study, patients with lower LVEF had the lower somatic domain score, as well as those with a higher NYHA class.

In previous papers, decreased systolic pressure, higher heart rate, and hyponatremia were associated independently with the risk of death or decreased QoL [53]. We did not observesuch associations in our study.

### 4.3. Iron Metabolism

In patients with HF, iron metabolism is one of the key issues. Iron is a microelement involved in the biochemical pathways of tissue metabolism [62]. The essential role of iron can be considered as taking part in transport, storage and usage of oxygen [63]. The deficiency of iron could be one of the most common comorbidities of HF, being a consequence of impaired erythropoiesis [64,65]. Cardiac myocytes have high energy requirements and are, therefore, particularly sensitive to iron restriction [66]. Moreover, iron deficiency (ID) also affects the functioning of skeletal muscle by impairing energetic metabolism [67]. Thus, the 2016 European Society of Cardiology (ESC) guidelines for the diagnosis and treatment of acute and chronic HF recommended that all patients with HF should be tested for ID [10].

Interestingly, the serum ferritin levels, one of the most commonly used laboratory parameter in assessing iron status worldwide, tend to increase in inflammatory and chronic diseases and HF is a perfect example of such illnesses [68]. Nonetheless, the commonly accepted criteria for detecting ID in this population are: serum ferritin < 100 μg/L (identifying absolute ID) or serum ferritin 100–299 μg/L in combination with transferrin saturation (T_sat_) < 20% (identifying functional ID) [69,70]. One must emphasize that neither serum iron nor serum transferrin (or TIBC) alone is reliable and sufficient for assessing iron status in HF patients. The ID prevalence in HF patients using standard definition ranges from 33% to 74% [64,71,72,73,74,75,76,77,78,79,80,81,82]. ID is more prevalent in decompensated (65–83%) [63,71,81,83,84] vs. stable HF patients (34–65%) [64,72,73,74,75,76,77,78,79,80,85,86,87]. The presence of ID is associated with decreased HRQoL as assessed using MLHFQ [74,88]. However, we used another questionnaire to assess HRQoL (WHOQoL-BREF). Moreover, we did not assess ID directly but only individual iron metabolism parameters.

### 4.4. Other Biochemical Parameters

Literature data have suggested the relationship between lipid profile and HRQoL. In our study, a higher total cholesterol, triglycerides and LDL levels were associated with better HRQoL. Previously, researchers have suggested that in hypertensive patients a decrease in LDL-C or an increase in HDL-C were independently associated with an increase in the HRQoL among middle-aged or older adults [89]. By contrast, other studies have shown that higher HRQoL in the EQ-5D questionnaire in centenarians was associated with higher levels of total cholesterol, triglycerides as well as LDL-C and HDL-C.A similar relationship was found between the total cholesterol level or LDL-C and HRQoL assessed in the EQ-VAS questionnaire [90]. However, that study [90] comprised much older subjects without HF.

### 4.5. Limitations

This study was conducted in a relatively small sample at a single medical center. This has potential limitations on being able to generalize the results. Further studies performed within a larger group of patients using different HRQoL instruments would be beneficial. The study reveals differences between patients with low and high HRQoL; however, it does not allow the determination of a casual relationship between QoL and the analyzed parameters.

## 5. Conclusions

The somatic domain of WHOQoL-BREF in patients with HFrEF is related to the patient’s clinical status (NYHA class, LVEF, and iron status). HRQoL was not associated with age and gender, or comorbidities. The routine evaluation of clinical, biochemical, and echocardiographic parameters may disclose lower HRQoL in HFrEF patients. HRQoL assessment in HFrEF patients is important in everyday practice and can identify patients needing a special intervention. More studies are required to develop a comprehensive program for assessing HFrEF patients.

## Figures and Tables

**Table 1:** Baseline characteristics of the study population (mean values and standard deviation, median and interquartile range or number and %).

Parameter (n = 152)	Value
Age [years]	57 (48–62)
Men	124 (81.6%)
Somatic QoL domain score [0–100]	50 (42.9–57.1)
Psychological QoL domain score [0–100]	66.7 (58.3–70.8)
Social QoL domain score [0–100]	75.0 (66.7–91.7)
Environmental QoL domain score [0–100]	71.9 (62.5–81.2)
Total QoL [0–400]	265.2 (239.7–285.7)
BMI [kg/m^2^]	28.1 (24.9–32.1)
IHD etiology	73 (48.0%)
HF exacerbation	40 (26.3%)
SBP on admission [mmHg]	112.3 ± 18.9
DBP on admission [mmHg]	70 (68–80)
HR on discharge [beats per minute]	72.0 (65–80)
**Comorbidities**	
DM	42 (27.6%)
COPD	12 (7.9%)
CKD	24 (15.8%)
Hypertension	80 (52.6%)
AF	62 (40.8%)
**NYHA class**	
I	3 (2.0%)
II	62 (40.8%)
III	71 (46.7%)
IV	16 (10.5%)
I–II	65 (42.8%)
III–IV	87 (57.2%)
**Biochemical parameters**	
BNP [pg/mL]	509.8 (213.1–869.7)
TSH [mIU/L]	1.82 (0.98–3.12)
Uric acid [µmol/L]	454 (358–554)
Creatinine [µmol/L]	95.0 (80.0–114.1)
eGFR [mL/min]	72.5 ± 23.7
Na^+^ [mmol/L]	140.0 (138.0–142.0)
K^+^ [mmol/L]	4.30 ± 0.42
hsCRP [mg/L]	4.0 (4.0–6.0)
Fasting glucose [mmol/L]	5.8 (5.3–6.7)
Serum protein [g/L]	71.5 (67.2–75.6)
Serum albumin [g/L]	40.3 (37.8–43.4)
CholT [mmol/L]	4.14 (3.37–4.95)
TG [mmol/L]	1.35 (1.05–1.80)
LDL [mmol/L]	2.48 (1.87–3.25)
HDL [mmol/L]	1.13 (0.93–1.41)
Hgb [mmol/L]	8.9 (8.3–9.6)
Serum iron [µmol/L]	15.8 ± 7.1
TIBC [µmol/L]	63.0 (56.1–70.2)
Transferrin saturation [%]	25.4 ± 12.1
Ferritin [ng/mL]	129.9 (61.8–218.3)
GNRI score	113.3 ± 12.2
High nutritional risk (GNRI <82)	0
Intermediate nutritional risk (GNRI ≥82 and <92)	4 (3.7%)
Low nutritional risk (GNRI ≥92 and ≤98)	5 (4.6%)
No nutritional risk (GNRI >98)	99 (91.7%)
**Echocardiographic parameters**	
LVEF [%]	22 (20–30)
**Medications (n)**	
Loop diuretics [%]	143 (94.1%)
Thiazides [%]	23 (15.1%)
β-blocker [%]	147 (96.7%)
ACEI/ARB [%]	98 (64.5%)
ARNI [%]	37 (24.3%)
MRA [%]	132 (86.8%)
Ca-blocker [%]	7 (4.6%)
Statin [%]	96 (63.2%)

Abbreviations: BMI—body mass index, IHD—ischemic heart disease, SBP—systolic blood pressure, DBP—diastolic blood pressure, HR—heart rate, DM—diabetes mellitus, COPD—chronic obstructive pulmonary disease, CKD—chronic kidney disease, AF—atrial fibrillation (paroxysmal, permanent or persistent),NYHA—New York Heart Association Classification, Na^+^ sodium concentration, BNP—B-type natriuretic peptide, eGFR—estimated glomerular filtration rate, K^+^—potassium concentration, hsCRP—high-sensitivity C-reactive protein, CholT—total cholesterol, TG—triglycerides, LDL—low-density lipoprotein, HDL—high-density lipoprotein, Hgb—hemoglobin, TIBC—total iron—binding capacity, GNRI—Geriatric Nutritional Risk Index, LVEF—left ventricular ejection fraction, QoL—quality of life, ACEI—angiotensin-converting enzyme inhibitor, ARB—angiotensin receptor blocker, MRA—mineralocorticoid receptor antagonist.

**Table 2:** Comparison of patients with the highest and the lowest score in the somatic domain of the World Health Organization’s Quality of Life Instrument–Short Version (WHOQoL-BREF) questionnaire.

Characteristics	Group 1 The Highest Quality of Life Group (Somatic Domain Score > 55) (n = 47)	Group 3 The Lowest Quality of Life Group (Somatic Domain Score < 50) (n = 58)	*p*
Age [years]	56.0 (46–62)	56.5 (49–62)	0.60
Men	39 (83.0%)	47 (81.0%)	0.99
Somatic QoL domain score [0–100]	64.3 (57.1–67.9)	42.9 (35.7–46.4)	<0.0001
Psychological QoL domain score [0–100]	66.7 (62.5–79.2)	62.5 (58.3–70.8)	0.004
Social QoL domain score [0–100]	83.3 (75.0–100)	66.6 (58.3–83.3)	<0.0001
Environmental QoL domain score [0–100]	75.0 (65.6–87.5)	68.8 (59.4–75.0)	0.001
Total QoL [0–400]	288.2 (267.6–317.3)	244.6 (219.5–263.1)	<0.0001
BMI [kg/m^2^]	28.7 (26.0–32.3)	27.1 (23.8–31.7)	0.08
IHD etiology	18 (38.3%)	31 (53.4%)	0.12
HF exacerbation	7 (14.9%)	17 (28.3%)	0.16
SBP on admission [mmHg]	115.0 ± 17.9	112.5 ± 19.6	0.50
DBP on admission [mmHg]	70 (68–80)	70(68–80)	0.81
HR on discharge [beats per minute]	70 (65–80)	72.5 (66–80)	0.29
**Comorbidities**			
DM	14 (29.8%)	20 (34.5%)	0.66
COPD	1 (2.1%)	8 (13.8%)	0.08
CKD	7 (14.8%)	11 (19.0%)	0.74
Hypertension	20 (42.6%)	28 (48.3%)	0.56
AF	15 (31.9%)	22 (37.9%)	
**NYHA class**			
I	3 (6.4%)	0	0.17
II	29(61.7%)	19 (32.8%)	0.003
III	15 (31.9%)	32 (55.1%)	0.02
IV	0	7 (12.1%)	0.04
I–II	32 (68.1%)	19 (32.8%)	0.0003
III–IV	15 (31.9%)	39 (57.3%)	
**Biochemical parameters**			
BNP level [pg/mL]	326.2 (162.2–687.9)	578.3 (209.6–1124)	0.06
TSH [mIU/L]	1.50 (0.98–3.07)	1.52 (0.96–2.73)	0.99
Uricacid [µmol/L]	424 (318–534)	492 (383–559)	0.03
Creatinine [µmol/L]	89.0 (79.0–107.0)	100.2 (83.0–123.0)	0.08
eGFR	77.3 ± 23.2	68.7 ± 21.2	0.052
Na^+^ [mmol/L]	140.0 (138.0–141.0)	139.5 (137.0–142.0)	0.44
K^+^ [mmol/L]	4.45 ± 0.39	4.21 ± 0.43	0.004
hsCRP [mg/L]	4.0 (2.6–5.6)	4.0 (3.2–6.6)	0.61
Fastingglucose [mmol/L]	5.8 (5.3–7.0)	6.1 (5.3–6.7)	0.92
Serum protein [g/L]	73.1 (69.5–77.9)	71.8 (67.6–75.5)	0.21
Serum albumin [g/L]	41.8 (40.0–44.0)	40.0 (38.0–43.4)	0.15
CholT [mmol/L]	4.61 (3.82–5.42)	4.14 (3.20–4.79)	0.01
TG [mmol/L]	1.48 (1.27–2.13)	1.18 (0.91–1.57)	0.006
LDL [mmol/L]	2.99 (2.38–3.60)	2.40 (1.80–2.92)	0.001
HDL [mmol/L]	1.15 (1.00–1.44)	1.20 (0.92–1.54)	0.96
Hgb [mmol/L]	9.2 (8.6–9.5)	8.8 (8.2–9.7)	0.24
Serum iron [µmol/L]	17.6 ± 7.0	15.1 ± 6.8	0.08
TIBC [µmol/L]	57.7 (52.7–68.6)	64.9 (58.5–68.2)	0.02
Transferrinsaturation [%]	29.7 ± 12.5	23.7 ± 11.1	0.01
Ferritin [ng/mL]	162.8 (76.2–255.5)	126.0 (60.3–209.4)	0.17
**Nutritional parameters**			
GNRI	117.8 ± 12.4	112.3 ± 11.4	0.045
**Echocardiographic parameters**			
LVEF [%]	25 (20–30)	20 (15–25)	0.003
**Medications (n)**			
Loopdiuretics	42 (89.4%)	56 (96.6%)	0.29
Thiazides	4 (8.5%)	12 (20.7%)	0.16
β-blocker	45 (95.7%)	58 (100%)	0.90
ACEI/ARB	28 (59.6%)	41 (70.7%)	0.23
ARNI	13 (28.3%)	13 (22.4%)	0.49
MRA	38 (80.9%)	52 (89.6%)	0.45
Ca-blocker	2 (4.3%)	1 (1.7%)	0.85
Statin	29 (61.7%)	38 (65.5%)	0.75

Abbreviations: BMI—body mass index, IHD—ischemic heart disease, SBP—systolic blood pressure, DBP—diastolic blood pressure, HR—heart rate, DM—diabetes mellitus, COPD—chronic obstructive pulmonary disease, CKD—chronic kidney disease, AF—atrial fibrillation (paroxysmal, permanent or persistent),NYHA—New York Heart Association Classification, Na^+^—sodium concentration, BNP—B-type natriuretic peptide, eGFR—estimated glomerular filtration rate, K^+^—potassium concentration, hsCRP—high-sensitivity C-reactive protein, CholT—total cholesterol, TG—triglycerides, LDL—low-density lipoprotein, HDL—high-density lipoprotein, Hgb—hemoglobin, TIBC—total iron-binding capacity, GNRI—Geriatric Nutritional Risk Index, LVEF—left ventricular ejection fraction, ACEI—angiotensin—converting enzyme inhibitor, ARB—angiotensin receptor blocker, MRA—mineralocorticoid receptor antagonist.

## Data Availability

Not applicable.
